# Nitrate of Silver in Erysipelas

**Published:** 1846-11

**Authors:** 


*Nitrate of Silver in Erysipelas.*—M. Jobert recommends the application of nitrate of silver in erysipelas, in the form of ointment rather than of the caustic itself. He gives three formulæ, of various strengths, for preparing this ointment. To 32 parts of lard, the strongest ointment has 12 parts of nitrate of silver, the next has 8 parts, whilst the weakest has only 4.—*Ibid., from Gazette des Hôpitaux.*

